# Bilateral Ocular ischemia in a patient with Takayasu arteritis

**DOI:** 10.22336/rjo.2026.45

**Published:** 2026

**Authors:** Neha Neha, Nripen Gaur, Lokesh Rana, Chakit Kumar

**Affiliations:** 1 Department of Ophthalmology, All India Institute of Medical Sciences, Bilaspur, Himachal Pradesh, India; 2 Department of Radiodiagnosis, All India Institute of Medical Sciences, Bilaspur, Himachal Pradesh, India

**Keywords:** Takayasu arteritis, Ocular Ischemic Syndrome, vision loss, retinal ischemia, TA = Takayasu Arteritis, OIS = Ocular Ischemic Syndrome, ESR = Erythrocyte Sedimentation Rate, CRP = C-Reactive Protein, ANA = Antinuclear Antibody, CT = Computed Tomography, ACR = American College of Rheumatology, RE = Right Eye, LE = Left Eye, FFA = Fundus fluorescein angiography, CNP = Capillary Non-Perfusion, cDMARDS = Conventional Disease-Modifying Antirheumatic Drugs

## Abstract

**Objective:**

To describe a rare case of bilateral ocular ischemia as the initial presentation of Takayasu arteritis and highlight the role of early recognition and treatment in preventing irreversible vision loss.

**Methods:**

A woman in her 50s presented with a gradual, progressive diminution of vision, initially in the left eye and later involving the right. Comprehensive ophthalmic evaluation, systemic examination, fundus fluorescein angiography, and CT angiography of the head and neck were performed. Laboratory investigations, including ESR, CRP, and ANA, were obtained. The American College of Rheumatology (ACR) criteria were applied to support the diagnosis.

**Results:**

The patient demonstrated severe ocular ischemic features, including attenuated retinal vessels, disc neovascularization, and intumescent cataract in the left eye. Systemic examination revealed feeble brachial and radial pulses with a significant inter-arm blood pressure difference. CT angiography showed narrowing of the right common carotid artery and non-opacification of both subclavian arteries. Laboratory findings revealed elevated ESR and CRP, with a positive ANA. Based on clinical, radiologic, and laboratory features, a diagnosis of bilateral ocular ischemia secondary to Takayasu arteritis was made. The patient was treated with oral corticosteroids (60 mg/day, tapered) and azathioprine. At 4 weeks, visual acuity improved to 6/36 in the right eye, while no improvement was noted in the left eye.

**Discussion:**

Our case highlights bilateral ocular ischemia as an uncommon but vision-threatening manifestation of Takayasu arteritis. Ocular findings such as retinal vascular attenuation, disc neovascularization, delayed choroidal filling, and peripheral capillary non-perfusion may precede the diagnosis of systemic vasculitis. Recognition of associated systemic signs, including pulse deficits and inter-arm blood pressure discrepancies, facilitated timely diagnosis. Prompt initiation of corticosteroid and immunosuppressive therapy led to visual improvement in the right eye, underscoring the importance of early diagnosis and multidisciplinary management to prevent irreversible visual loss and systemic vascular complications.

**Conclusion:**

Bilateral ocular ischemia may be the first manifestation of Takayasu arteritis. This case underscores the importance of a high index of suspicion in patients with unexplained ischemic ocular features. Early systemic evaluation and prompt initiation of immunosuppressive therapy are essential to preserve vision and prevent further vascular complications.

## Introduction

Takayasu arteritis is a rare autoimmune condition characterized by granulomatous vasculitis, primarily affecting medium and large-sized arteries. It is more common among women and is most frequently observed in individuals of Asian or Mexican descent. This condition results in the fibrous thickening of arterial walls, leading to various vascular obstructions and ischemic changes. The initial symptoms of Takayasu arteritis are often nonspecific. As the disease progresses, signs of arterial occlusion, aneurysm formation, and vascular pain become more evident. One significant complication of Takayasu arteritis is ocular ischemic syndrome, which can result in blindness.

## Case presentation

A woman in her 50s presented to us with chief complaints of gradual, progressive diminution of vision beginning with the left eye, and eventually involving both eyes. She had a history of a severe headache on the left side and unintentional weight loss in the last 6 months. She denied any history of arthralgia, claudication, convulsion, or syncope. She had multiple outpatient visits in the past 1 year due to her ongoing visual symptoms. On ocular examination, her vision in RE was counting fingers at 2 meters and no perception of light in LE. Intraocular pressure was 8 mmHg in RE and 10 mmHg in LE. An afferent pupillary defect was present. On slit lamp examination, RE revealed a quiet anterior chamber with no iris vascularization. In LE, an intumescent total cataract was present. On fundus examination, neovascularization was present on the disc, and retinal vessels were attenuated in RE. On systemic examination, both brachial and radial pulses were feeble. Blood pressure readings were 130/80 mmHg on the right side and 80/60 mmHg on the left side.

### Investigations

**Fig. 1:**
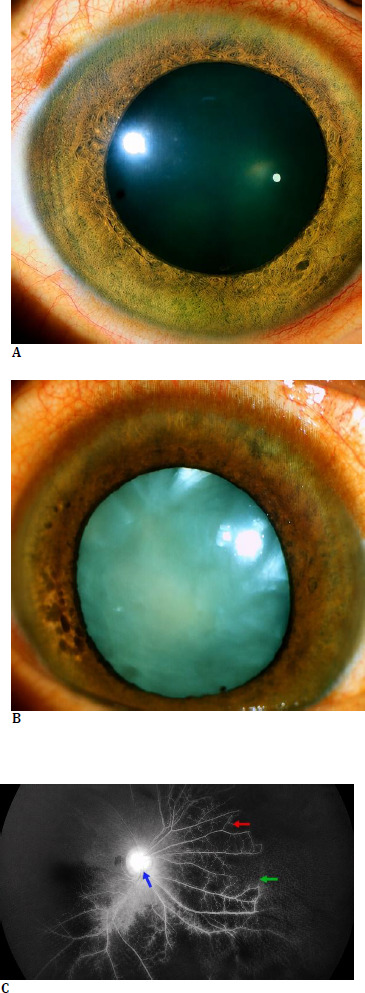
A. Anterior segment image of the right eye (RE) showing a quiet anterior chamber and absence of iris vascularization; B. Anterior segment image of the left eye (LE) showing intumescent total cataract; C. Fundus Fluorescein angiography (late phase) - green arrow: microaneurysm, red arrow: arteriovenous shunt, blue arrow: disc collaterals

**Fig. 2:**
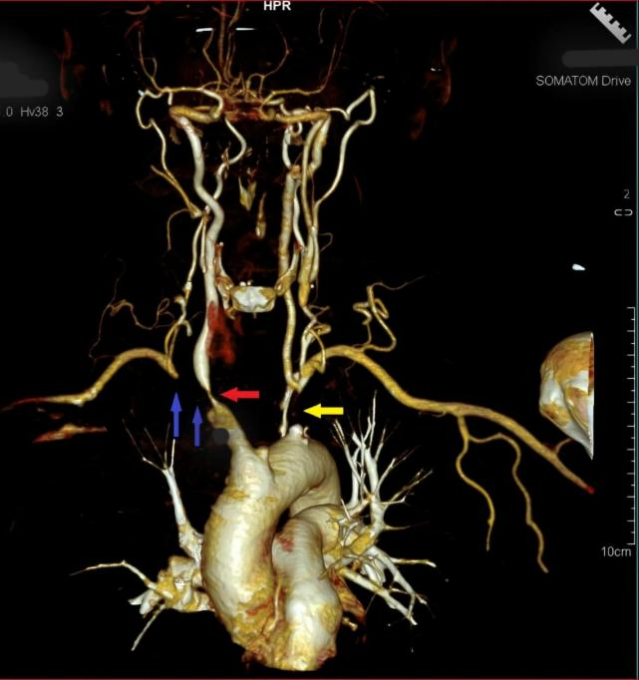
Volume rendered image of CT head and neck angiography. Red arrow: significant narrowing of the right common carotid artery, blue arrow: non-opacification of the proximal right subclavian artery, yellow arrow: non-opacification of the proximal left subclavian artery

Ultrasound examination of LE showed an attached retina. Fundus fluorescein angiography in RE revealed delayed choroidal filling, disc collaterals (Fig. 1C, blue arrow), arteriovenous shunts (Fig. 1C, red arrow), microaneurysms (Fig. 1C, green arrow), and peripheral capillary non-perfusion (CNP) areas. Both the erythrocyte sedimentation rate (ESR) and C-reactive protein (CRP) levels were elevated. Liver, renal, and thyroid function tests were normal. Serology for antinuclear antibodies (ANA) was positive. A volume rendered image of CT angiography of the head and neck indicated significant narrowing of the right common carotid artery (Fig. 2, red arrow), as well as non-opacification of the proximal right subclavian artery (Fig. 2, blue arrow) and the proximal left subclavian artery (Fig. 2, yellow arrow).

### Diagnosis

Based on medical history, examination, and CT head and neck angiography findings, the patient was diagnosed with bilateral ocular ischemia secondary to Takayasu arteritis.

### Treatment

The patient was prescribed oral steroids at a dosage of 60 mg per day, with a tapering schedule of 10 mg every 10 days. In addition to the oral steroids, the patient was started on conventional disease-modifying antirheumatic drugs (cDMARDs), specifically azathioprine at a dosage of 50 mg.

### Outcome and follow-up

At 4-week follow-up, her vision improved to 6/36 in RE. In LE, there was no improvement in visual acuity.

## Discussion

Takayasu arteritis is a granulomatous polyarteritis affecting medium and large arteries. The incidence of Takayasu arteritis ranges from 0.3 to 3.3 million per year, and the prevalence ranges from 4.7 to 360 million per year [1]. The pathophysiology of Takayasu arteritis involves a complex interplay among immune-mediated processes, vascular remodeling, and genetic factors [2]. The American College of Rheumatology (ACR) criteria include: age < 40 years, claudication of the extremities, decreased brachial artery pulse, a blood pressure difference of >10 mmHg between arms, a bruit over the subclavian arteries or the aorta, and an arteriographic abnormality. At least 3 of 6 criteria should be present for the diagnosis of Takayasu arteritis. Clinical manifestation of TA is classified either into the hypertensive or hypoperfusion group [3]. Manifestations in the hypertensive group are due to the involvement of the renal artery or suprarenal aorta, leading to hypertension. In the hypoperfusion group, manifestations result from occlusive arteritis of the aorta and its branches, leading to ischemic ocular symptoms.

In our case, the diagnosis was based on ACR guidelines, with our patient fulfilling three out of the six criteria: decreased brachial artery pulse, a blood pressure difference of more than 10 mmHg between the arms, and arteriographic abnormalities. The ischemic ocular features observed in our patient were attributable to the hypoperfusion phase. They included retinal vascular attenuation, arteriovenous shunts, microaneurysms, and disc neovascularization, which are well-described ocular findings in Takayasu arteritis [4]. Cataracts in Takayasu arteritis could be either due to ocular ischemia or complications of steroid therapy. In our case, it was attributed to ocular ischemia as the patient was not a known case of Takayasu arteritis, and steroids were not started earlier. Pallangya et al. have reported a similar case in which bilateral ocular ischemia was the presenting manifestation of Takayasu arteritis [5].

## Conclusion

Management of Takayasu arteritis involves the use of steroids and conventional disease-modifying antirheumatic drugs (cDMARDS) as first-line therapy in all active Takayasu arteritis patients. In our case, the patient was started on oral prednisolone and azathioprine. There was a marked improvement in RE vision after 4 weeks. This case demonstrates ocular ischemia as a presenting feature of Takayasu arteritis and emphasizes the need for a high index of suspicion in such cases. Early diagnosis and treatment are necessary to prevent further complications of Takayasu arteritis.

## Presentation at a meeting

Nil.
